# Modeling and Experimental Verification of Material Welding Characteristics for Low Current Switching Devices

**DOI:** 10.3390/ma13173666

**Published:** 2020-08-19

**Authors:** Xu Zhang, Wanbin Ren

**Affiliations:** School of Electrical Engineering and Automation, Harbin Institute of Technology, Harbin 150001, China; hitee_zhangxu@163.com

**Keywords:** material welding, threshold welding current, welding force, welding area, tensile strength

## Abstract

Material welding failure considerably influences the electrical lifetime and reliability of low current switching devices. However, relevant studies on methods for calculating the threshold welding current and welding area under milli-Newton scale load forces are very limited. In this paper, the welding characteristics of metal material, including the threshold welding current, welding area and welding force are studied by using theoretical calculations and experiments. The comparison between the theoretical calculation and experimental results shows the accuracy of the built model. Further, the effects of mechanical load force and load current on welding force and welding area of representative metal materials are investigated. It is found that the anti-welding ability of metal materials depends not only on the exerted load force and current, but also the electrical resistivity, the thermal conductivity, the tensile strength, and the melting temperature of the materials.

## 1. Introduction

Low current switching devices, such as relays or contactors, play an important role in domestic and industrial applications as they have the advantages of low conduction loss and high off-isolation. They are also low-cost, and more thermally and electromagnetic compatibility (EMC)-robust compared with solid-state switches [1,2]. With the rapid development of solar power plants and battery storage systems, there has been an increasing demand for traditional controlling/switching devices, which must withstand transiently high overload currents or surge currents [3,4].

Material welding can occur if a high enough current passes through closed contacts and causes the contact spot to melt [5,6,7]. The excessive welding force for switching electrode in such a state can lead to the electrodes failing to open and must therefore be addressed. Practically, the information of a weld is a complex function of the circuit, depending on factors like whether or not arcing occurs, the physical properties of the metal material, the microscopic surface roughness and the design of the structures in which the contacts operate [8,9,10,11].

Due to the fact that material welding failure always occurs in high voltage circuit breakers, much effort has been devoted, in the last decade, to the investigation of material welding characteristics in a vacuum interrupter [12,13,14]. With the use of the classical Kohlrausch Equation, Slade [15] developed an easily usable equation for the threshold welding current, *I*_weld_, for a single region of contacts as a function of the applied load force *F*, and for a current pulse of a few milliseconds, see Equation (1):

(1) $$I_{\mathrm{weld}}=\frac{2U_{m}\sqrt{F}}{{\left[{\left\{0.1\pi H_{0}\rho_{0}[1+\frac{2}{3}\alpha (T_{1}-T_{0})]\right\}}^{2}+1.78\times 10^{-6}U_{m}{}^{2}\right]}^{1/2}}$$

where *I*_weld_, is in A, *F* in N. *U_m_* is the metal material’s melting voltage in V, which is the minimum voltage required for the contact materials to reach the melting point by the heat generated by resistance. *ρ*_0_ is the resistivity in Ω·mm and *H*_0_ is the hardness of metal material in N·mm^−2^ at ambient temperature *T*_0_, which is in K. *T*_1_ is a temperature close to, but less than the metal material’s melting temperature *T_m_* and *α* is the temperature coefficient of resistivity in K^−1^.

Kharin et al. [16] established a mathematical model of material welding for an AC current half-wave and proposed a method of calculating the welding area considering the softening contact zone. Tslaf [17] proposed a thermal conduction model for cross bar contacts, and pointed out that the welding resistance ability of metal materials is determined by the coefficient *η_w_*, which directly depends on the melting temperature, the resistivity, and the heat conductivity.

At the same time, many experimental investigations have been presented for better understanding the welding characteristics of materials. Borkowski et al. [18] reported an automated test stand, which can measure contact voltage and welding force synchronously for evaluating the welding performance of materials carrying a high load current. Slade et al. [12] experimentally studied the effect of short circuit currents on the welding of closed contacts in vacuum circuit breakers for load forces of 1.3–2.1 kN and a current of 20 kA, and concluded that the increase of load force and/or the reduced current duration could substantially inhibit the material welding. Chalyi et al. [19] examined the effect of short-duration pulsed currents on the welding strength of closed electrodes for load forces of 100 N and 300 N.

However, the force of closed contacts in low current switching devices is usually on a milli-Newton scale, which results in the fact that most of these research findings are not suitable for the welding issues in the elastic deformation situation. Therefore, in this paper a mathematical model for calculating the temperature distribution of electrode materials resulting from the elastic contact load and the electrical current load is built. Next, the threshold value of the welding current, and the welding area of representative electrode materials (silver, copper, silver tin oxide and silver nickel alloy) are calculated and compared. Further, a novel test rig which can adjust the load current and mechanical load force flexibly is designed to measure the welding force and welding area simultaneously. The material tensile strength is also determined for the evaluation of welding resistance.

## 2. Mathematical Model 

### 2.1. Threshold Welding Current

**Figure 1:**
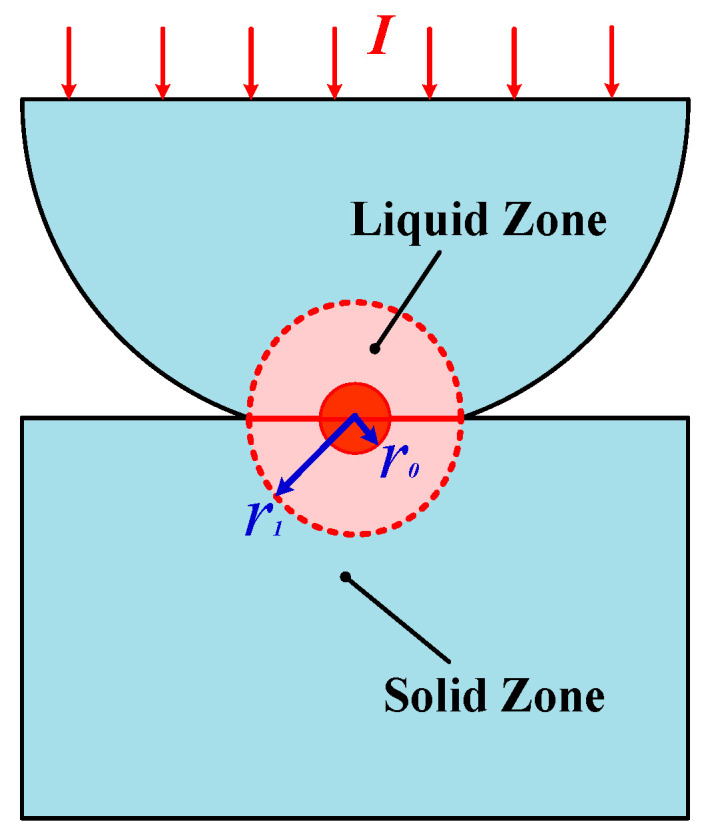
Schematic model of sphere-plane contact pair.

Figure 1 depicts an elastic hemisphere in contact with a flat surface to create a point contact for a normally closed contact pair, and the contact material is assumed to be homogeneous and isotropic. It can be considered that the contact surface in the area of current concentration is heated by a high-amperage current for long time periods to cause welding of the material, and the threshold welding current *I*_weld_ passing through this contact spot can be expressed as:

(2) $$I_{\mathrm{weld}}=\frac{U_{m}}{R}$$

where *U_m_* is the melting voltage of material, *R* is the contact resistance.

According to the classical Holm’s electrical contact theory, the contact resistance of two components with clean surfaces can be expressed as:

(3) $$R=\frac{\rho }{2r_{0}}$$

in which *ρ* is the resistivity of the metal material, *r*_0_ is the radius of the contact spot.

The load force *F_t_* of typical low current switching devices is on the order of tens of milli-Newton, therefore in the elastic deformation situation, the contact radius *r*_0_ is written as:

(4) $$r_{0}=\sqrt[{3}]{\frac{F_{t}}{E}a}$$

in which *E* is the modulus of elasticity and *a* is the radius of the hemisphere.

The resistivity *ρ* of electrode materials at temperature *T*_1_ higher than the ambient temperature *T*_0_ is:

(5) $$\rho =\rho_{0}[1+\frac{2}{3}\alpha (T_{1}-T_{0})]$$

in which *ρ*_0_ is the metal material’s bulk resistivity and *α* is the temperature coefficient of resistivity. If the blow-off force is considered, the total force holding the contacts together is [5]:

(6) $$F_{t}=F-4.45\times 10^{-7}I_{\mathrm{weld}}{}^{2}$$

Combining Equations (2)–(6), then the implicit expression of the threshold welding current *I*_weld_ is shown in Equation (7):

(7) $$I_{w}{}^{3}+4.45\times 10^{-7}\frac{a}{E}{\left(\frac{2U_{m}}{\rho_{0}[1+\frac{2}{3}\alpha (T_{1}-T_{0})]}\right)}^{3}I_{w}{}^{2}-\frac{Fa}{E}{\left(\frac{2U_{m}}{\rho_{0}[1+\frac{2}{3}\alpha (T_{1}-T_{0})]}\right)}^{3}=0$$

### 2.2. Heat Conduction Model

As shown in Figure 1, it is assumed that the initial contact area is a circular *a*-spot with a radius of *r*_0_, and the load current is concentrated in the contact zone. It can be considered that the contact zone melts and expands as a result of Joule heating when the load current is high enough. The expansion of the contact zone is accompanied by a decrease of heat production and an increase of heat conduction. As a consequence, the temperature distribution of the contact zone eventually reaches the thermal equilibrium state. Two distinguishing zones, that is liquid zone and solid zone, are expected to co-exist with an absolute boundary line. When the load current is cut off, the contact zone tends to weld with the cooling and solidification of molten metal. Material welding is a very complex dynamic physical process, involving the phase change of the material, the movement of fluid and heat transfer, so it is difficult to establish and solve a complete physical model. For the sake of simplicity, only the final thermal equilibrium state of electrode pairs is built to estimate the liquid zone, that is to say, the welding area. The system of interest and associated boundary conditions are described in relation to Figure 1.

We give the following assumptions, that is: (1) The melting zone is composed of two symmetrical hemispheres, and there is no heat exchange on the interface; (2) The expansion of melting zone stops when the contact temperature is invariable, and *r*_1_ is the radius of the boundary between liquid zone and solid zone, and the corresponding temperature here is the melting temperature *T_m_*. Further, the melting area π*r*_1_^2^ is defined as the mechanical contact area after expansion, and the load current is uniformly distributed over the contact area; (3) The deformation of heated material and further expansion of the contact area caused by the load force is not considered; (4) The radius of contact zone is far less than the side length of electrode, so the outer boundary of solid zone is taken as the infinity; (5) The welding zone equals to the melting zone.

**Figure 2:**
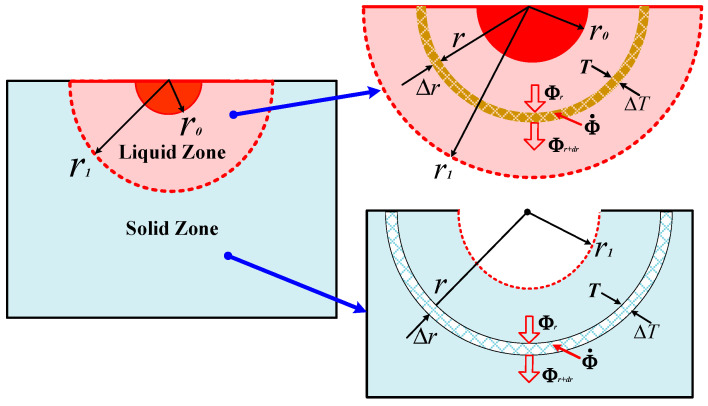
Hemispherical contact model for analytical calculation.

The model for analytical calculation is presented in Figure 2, and the partial differential equation of heat conduction of the hemispherical shell with thickness ∆*r* at radius *r* can be expressed as:

(8) $$\nabla \cdot (\lambda \nabla T)+\overset{\cdot }{\Phi }=0$$

where *λ* is the thermal conductivity, *T* is the temperature, and $\overset{\cdot }{\Phi }$ is the heat production of hemispherical shell. For simplicity, thermal conductivity *λ* is considered in these calculations to be constant.

Kharin concluded that the core of contact can be taken as the isothermal zone with the radius *r*_0_, which is same with the initial contact radius [20]. Therefore, Equation (8) in a spherical coordinate system can be revised into:

(9) $$\frac{d^{2}T}{dr^{2}}+\frac{2}{r}\frac{dT}{dr}+\frac{1}{\lambda }\overset{\cdot }{\Phi }=0$$

in which the heat production $\overset{\cdot }{\Phi }$ is given as follows:

(10) $$\overset{\cdot }{\Phi }=\{\begin{array}{c} \frac{I^{2}\rho (T)}{4\pi^{2}}\frac{1}{r_{1}{}^{4}},(r_{0}<r<r_{1})\\ \frac{I^{2}\rho (T)}{4\pi^{2}}\frac{1}{r^{4}},(r_{1}<r<\infty ) \end{array}$$

where *I* is the load current, *ρ*(*T*) is the electrical resistivity dependent on temperature *T*. The boundary conditions are taken as follows:

(11) $$\frac{dT}{dr}|\begin{array}{c} r=r_{0} \end{array}=0$$

(12) $$T_{1}|\begin{array}{c} r=r_{1} \end{array}=T_{2}|\begin{array}{c} r=r_{1} \end{array}=T_{m}$$

(13) $$T_{2}|\begin{array}{c} r=\infty \end{array}=0$$

(14) $$-\lambda \frac{dT_{1}}{dr}|\begin{array}{c} r=r_{1} \end{array}=-\lambda \frac{dT_{2}}{dr}|\begin{array}{c} r=r_{1} \end{array}$$

where *T_m_* is the melting temperature of materials.

**Figure 3:**
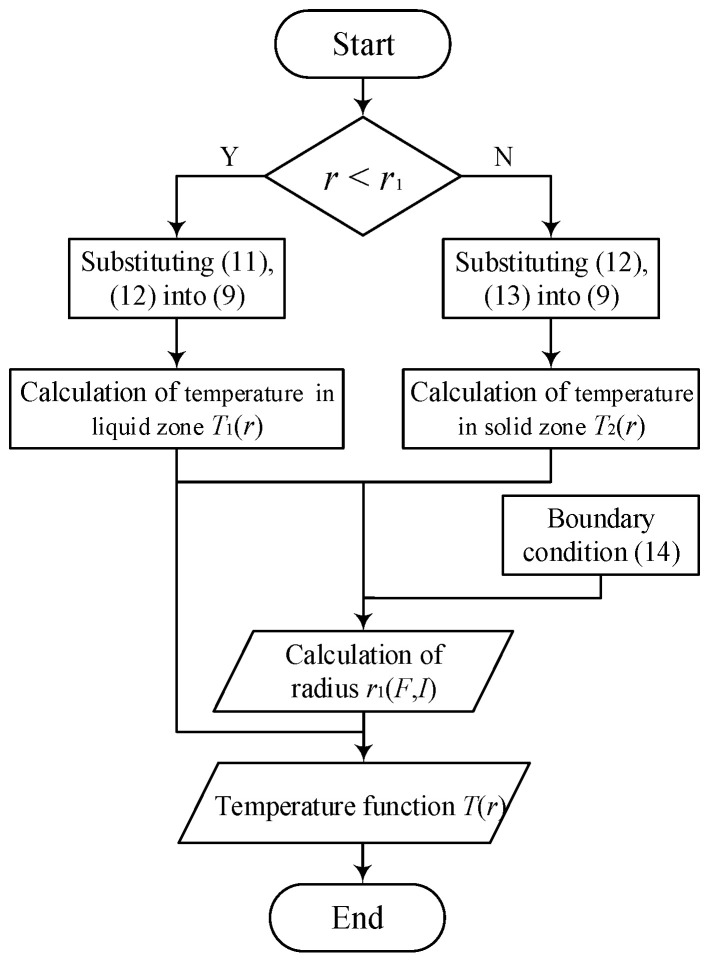
Flow chart of the whole calculation.

The temperature functions of the liquid zone and the solid zone *T*_1_(*r*) and *T*_2_(*r*) are calculated respectively by using the boundary conditions Equations (11)–(13). Further, the temperature functions *T*_1_(*r*) and *T*_2_(*r*) are substituted into Equation (14), then the welding radius *r*_1_ can be obtained by the bisection method, where the initial value of *r*_1_ is same as that of *r*_0_, and the iteration converges on a solution which is known to lie inside interval between *r*_0_ and *r_2_*, where the value of *r*_0_ is calculated by Equation (4) according to the known load force *F* and *r_2_* is taken as 50 × 10^−6^ m respectively. The desired accuracy is set to 1 × 10^−6^. Finally, the relationship between of *r*_1_, *F* and *I* is obtained, and then the temperature distribution function *T*(*r*) is obtained by using the intermediate variable *r*_1_(*F*, *I*). The flow chart of whole solution process is shown in Figure 3, which can be recommended as one of possible algorithms of the solution.

## 3. Calculation Results and Discussion

### 3.1. Threshold Welding Current

**Figure 4:**
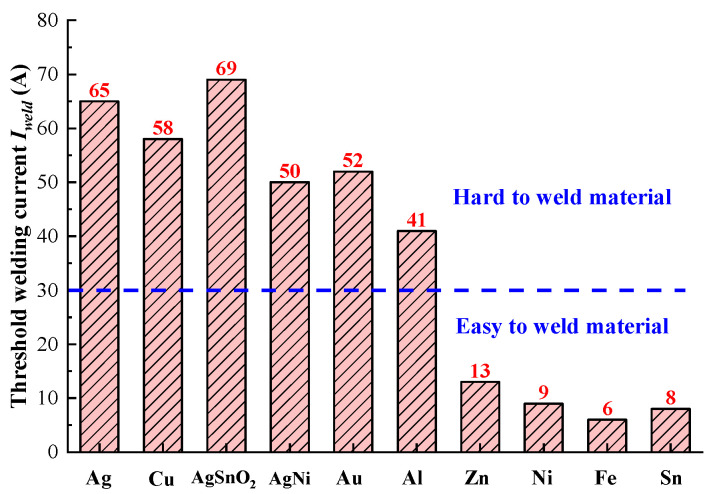
Threshold welding current for different materials (load force is 50 mN).

Table 1 shows the relevant physical properties for several representative metal materials. Taking the load force *F* as 50 mN, the radius of sphere *a* as 200 μm, then the threshold welding current *I*_weld_ for different materials are calculated and presented in Figure 4. We further define the material with a threshold welding current *I*_weld_ higher than 30A as the hard to weld material. As shown, the threshold welding currents *I*_weld_ of common used electrical contact materials silver, copper, silver tin oxide and silver nickel alloy are 65, 58, 69 and 50 A, respectively.

Therefore, they could be considered as the absolutely hard to weld materials, while Zn, Ni, Fe and Sn are classified as easy to weld materials. It should be noted that the anti-welding ability of a material is the comprehensive result of its physical properties, which are involved in the calculation model.

As expected, the electrical resistivity of hard to weld materials is uniformly lower than that of easy to weld materials. According to Equations (2) and (3), the threshold welding current is obtained from the ratio of melting voltage *U_m_* to contact resistance *R*, which is inversely proportional to the electrical resistivity *ρ*. Therefore, the low electrical resistivity *ρ* is known to be a major cause of high threshold welding current *I*_weld_ when the melting voltage *U_m_* is fixed.

**Figure 5:**
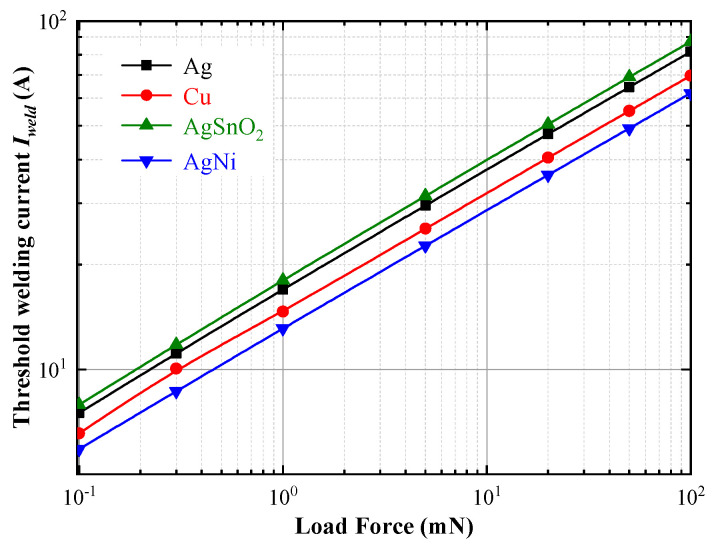
Variations in threshold welding current as a function of load force for Ag, Cu, AgSnO_2_ and AgNi.

Figure 5 illustrates the variation of threshold welding current *I*_weld_ as a function of load force for silver, copper, silver tin oxide and silver nickel alloy electrode materials. As shown in double logarithmic graph, the threshold welding current *I*_weld_ linearly increases when the load force varies from 0.1 mN to 100 mN, which correlates well with the findings of other researchers [15,22]. According to Equations (2)–(4), the expansion of contact radius resulted by higher load force leads to the decrease of contact resistance *R*, which corresponds to the elevation of threshold welding current *I*_weld_. The relationship between load force and threshold welding current *I*_weld_ indicates that the higher load force could improve the welding resistance of electrode materials.

### 3.2. Temperature Distribution

**Figure 6:**
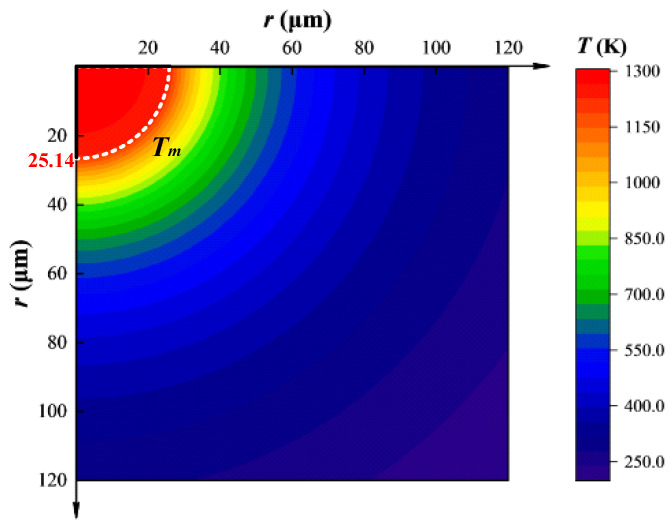
Temperature distribution contour (material: silver).

The calculation results of temperature distribution of silver for the load force of 50 mN and load current of 200 A is plotted in Figure 6. As shown, the temperature of the isothermal zone within the radius of 5.02 μm is the highest (1300 K). The white dotted line marked in the figure represents the melting temperature of silver (1234 K), which is also the boundary line between the liquid and solid zone. The corresponding radius *r*_1_ is 25.14 μm, and the associated welding area *A*_weld_ is calculated as 1.99 × 10^−3^ mm^2^. When the radius extends to 120 μm in the circumferential direction, the temperature drops substantially to 288 K, which is almost 80% of the maximum.

Kohlrausch showed that an increase in current density can raise the temperature for the contact zone [23]. Considering that the current density of liquid zone (*J_l_* = *I*/2π*r*_1_^2^) is much higher than that in solid zone (*J*_s_ = *I*/2π*r*^2^), it is easy to predict that the temperature in the liquid zone is higher. With the increase of the radius *r*, the current density decreases rapidly in the solid zone, but remains constant in the liquid zone, therefore a slight temperature variation appears in the liquid zone. It also results in the temperature of electrode dropping sharply in the solid zone, and almost no obvious variations in the liquid zone, as shown in Figure 6.

**Figure 7:**
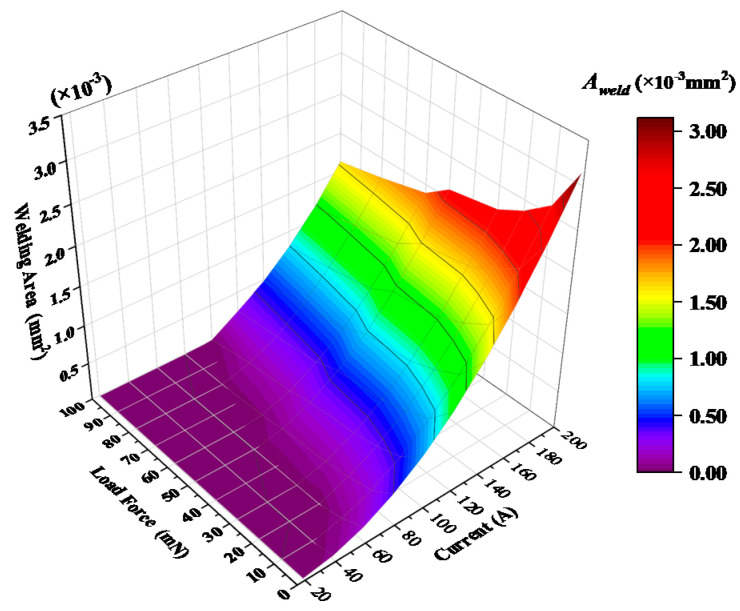
Variation in welding area as a function of load current and load force.

Figure 7 illustrates the contour map of calculated welding area of silver material under different load forces and currents. It is clearly observed that the critical threshold welding current and the resulted welding area relates closely with mechanical load forces and electrical currents. The welding area could reach to 3.13 × 10^−3^ mm^2^ when the load force is 1 mN and the current is 200 A. Also, the stronger load force leads to the reduction of welding area for the same load current and the higher current results into the increment of welding area for the same mechanical load force.

As known, the only reason for contact welding is the substantial Joule heat generated by the high current through constriction. The melting heat of electrode material directly relates to the heat production, which equals to the product of *I*^2^, contact resistance *R* and current duration *t*. Therefore, it is reasonably believed that the high load current could enhance the heat production and result in the strong welding phenomena. Similarly, the longer current duration *t* corresponds to the higher melting heat and associated stronger welding strength.

**Figure 8:**
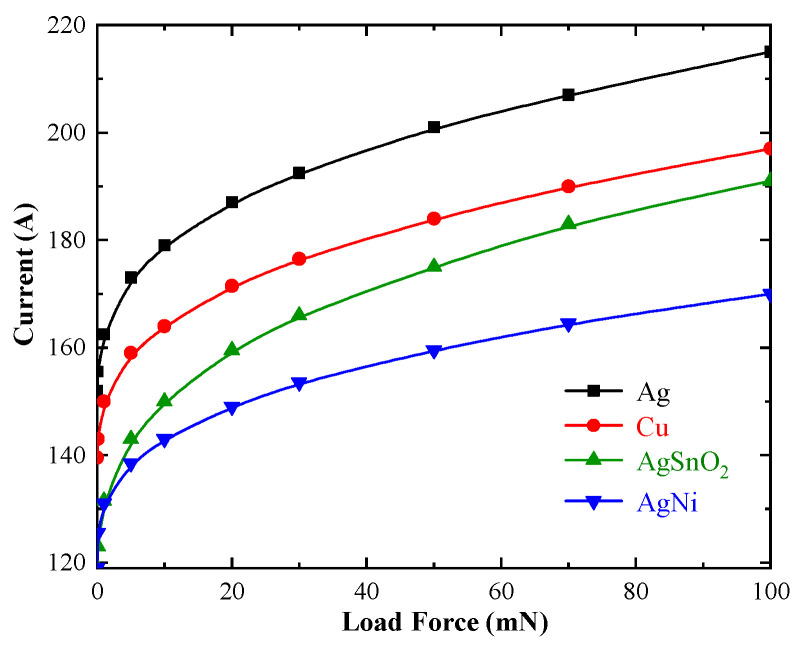
Variations in required load current as a function of load force for Ag, Cu, AgSnO_2_ and AgNi material at the same welding area of 2 × 10^−3^ mm^2^.

Figure 8 illustrates the variations in required load current as a function of load force for silver, copper, silver tin oxide and silver nickel alloy material at the same welding area of 2 × 10^−3^ mm^2^. It is noted that the required welding current increases sharply when the mechanical load force is lower than 10 mN, and the current continues to show an upward tendency with the increase of load force. When the load force is 100 mN, the required load currents for silver, copper, silver tin oxide and silver nickel alloy are 215, 197, 191 and 170 A, respectively. Moreover, it clearly indicates that silver material is the hardest to weld material. Admittedly, the welding characteristics ultimately depend on many parameters (electrical resistivity, thermal conductivity, tensile strength, melting temperature, etc.) of metal material. As known, the low electrical resistivity means the reduction of Joule heat, meanwhile the high thermal conductivity is beneficial to material heat transfer and the small tensile strength of materials is rather prone to result in weak welding.

## 4. Model Validation

### 4.1. Experimental Conditions

**Figure 9:**
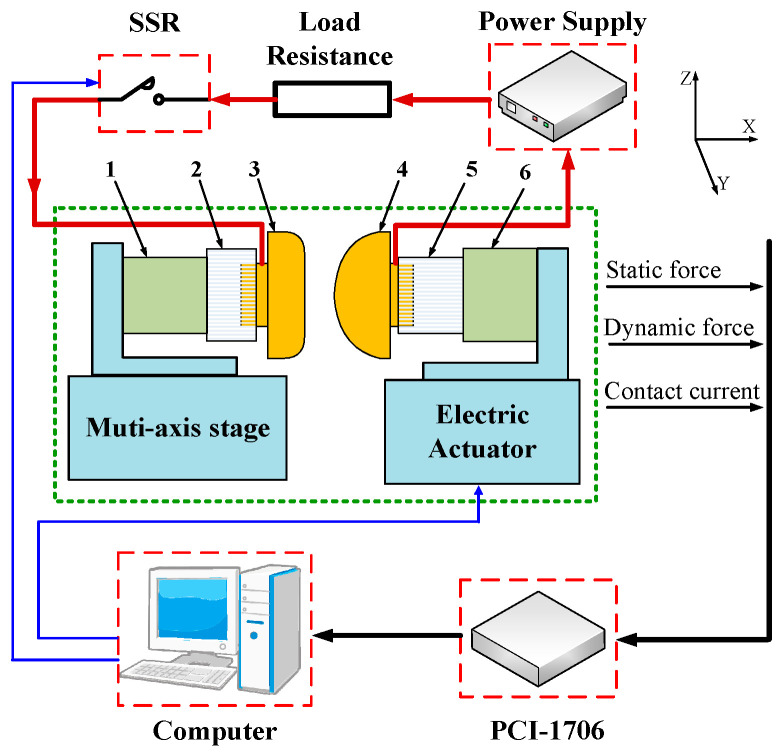
Schematic plot of the new designed test rig (1. Dynamic force transducer 2. Insulation block 3. Stationary contact 4. Movable contact 5. Flexible joint 6. Strain transducer).

An experimental evaluation method of welding characteristics for metal materials is designed in order to verify the built mathematical model mentioned above. A schematic diagram of the test rig is shown in Figure 9.

**Figure 10:**
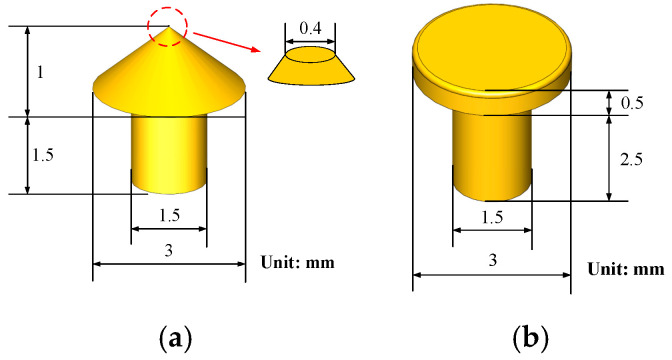
Rhino-3D pictures of samples. (**a**) Cone; (**b**) Plane.

The measurement of welding force is realized by a piezoelectric dynamic transducer (209C11, PCB, Depew, NY, USA) with an upper frequency limit of 30 kHz and a force resolution of 0.09 mN. The initial load force is measured by a strain transducer (FA404-2kg, FIBOS, Changzhou, China), which provides a resolution of 1 mN. The contact current is measured by a Hall current sensor, which has the resolution of 50 mA. The horizontal actuation of the movable contact is obtained by an electric actuator (RCA2, IAI, Shizuoka, Japan). All above signals including contact current, dynamic force and static load force are acquired by a commercial DAQ system (PCI1706, Advantech, Taipei, Taiwan), which has a measurement resolution of 16 bits and a sampling frequency of 250 kHz. The instrument is interfaced to an industrial computer through a PCI bus. The data acquisition and logging process are controlled by LabVIEW software. A scanning electron microscopy (Quanta FEG, FEI, Hillsboro, OR, USA) is used to characterize the welded surface morphology. The sample materials are selected as silver (99.99%), copper (99.99%), silver tin oxide (Ag: 99.9%, SnO_2_: 99%, 88/12) and silver nickel alloy (Ag: 99.9%, Ni: 99.9%, 90/10). The movable electrode is cone-shaped, and the stationary electrode is plane-shaped, and the Rhino-3D diagrams of the experimental samples are shown in Figure 10. The samples are degreased using alcohol and distilled water in an ultrasonic cleaner, dried, and carefully mounted in the test rig. The details of the experimental conditions are listed in Table 2.

### 4.2. Welding Trace and Associated Area

**Figure 11:**
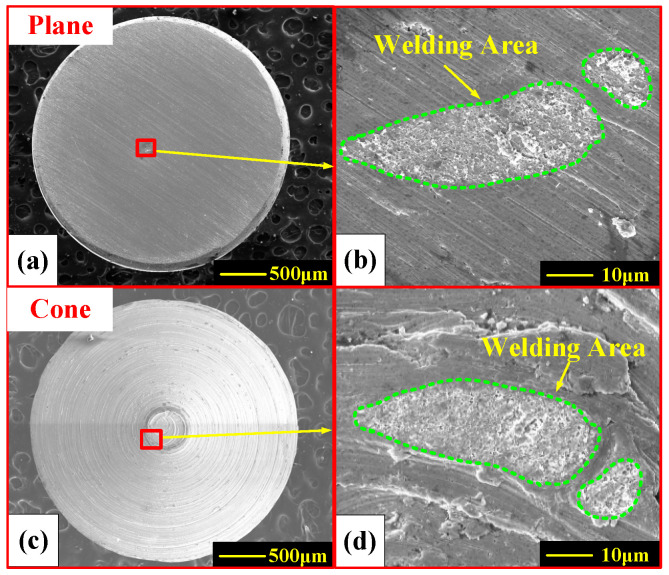
SEM pictures of electrodes after experiment (load current is 140 A and load force is 50 mN). (**a**) Stationary electrode. (**b**) Zoom view of (**a**). (**c**) Movable electrode. (**d**) Zoom view of (**c**).

Figure 11 shows the representative surface morphology of silver tin oxide electrode material for a breaking load current of 140 A and load force of 50 mN.

Zoomed-in views of the welded region are shown in Figure 11b,d and the welding traces in both figures are marked by green dotted lines. As shown, there are two clearly separated crescent-shaped welded traces. The SEM pictures are captured with a resolution of 1024 pixels × 768 pixels and the standard length of 10 μm in Figure 11b corresponds to avalue of 102 pixels. Thus, the length of a single pixel *l* is calculated as 10/102 = 9.804 × 10^−5^ mm, and the area of one single pixel is considered as the square of the pixel length *l*, that is, 9.612 × 10^−9^ mm^2^. The two welded traces correspond to 109,898 pixels and 18,307 pixels separately, hence, the total welding area *A*_weld_ = (109,898 + 18,307) × *a* = 1.232 × 10^−3^ mm^2^. The welding force is measured as 328 mN.

### 4.3. Experimental Results

**Figure 12:**
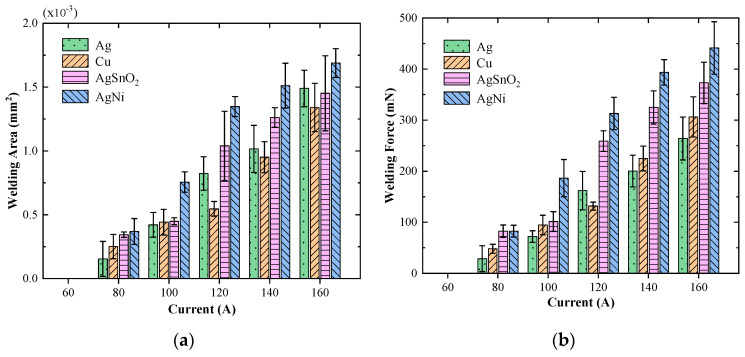
Variations in welding area and welding force as a function of current for Ag, Cu, AgNi and AgSnO_2_. (**a**) Welding area. (**b**) Welding force.

Figure 12 illustrates the variation of welding area and welding force as a function of load current of silver, copper, silver tin oxide and silver nickel alloy for load force of 50 mN. As shown, there is a clear trend that the average of welding area and welding force of the selected electrode materials increases monotonously with the increment of load current from 60 A to 160 A. The welding area of silver nickel alloy material is the largest at all current levels, reaching up to 1.77 × 10^−3^ mm^2^ and the associated welding force is 441 mN. There is no welding phenomenon when the load current drops to 60 A, so the threshold welding current of the abovementioned four metal electrode materials is estimated to lie between 60 A and 80 A.

**Figure 13:**
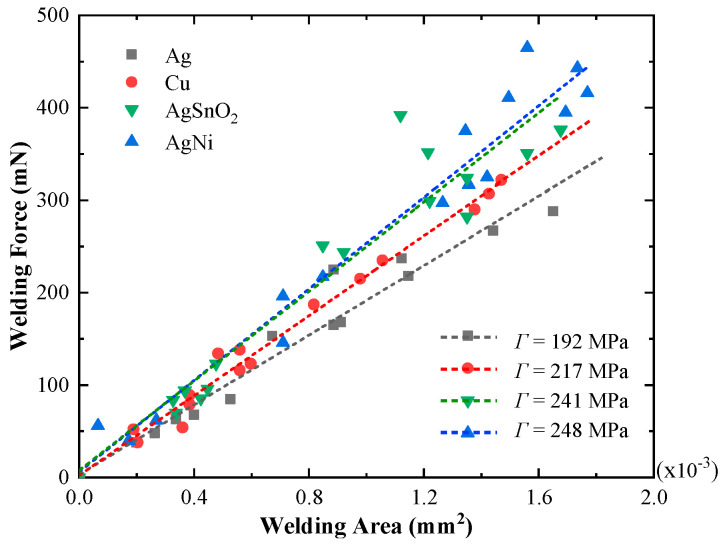
The relationship between welding area and welding force for Ag, Cu, AgSnO_2_ and AgNi. (load current ranges from 80 A to 160 A and load force is 50 mN).

The relationship between welding area and welding force of silver, copper, silver tin oxide and silver nickel alloy for constant load force of 50 mN is illustrated in Figure 13. As shown, all of the recorded welding areas are distributed within 2 × 10^−3^ mm^2^, and there is a clear trend that the average of welding force linearly increases with the increment of welding area. According to [5], the welding force *F_w_* is given by:

(15) $$F_{w}=\Gamma A_{\mathrm{weld}}$$

where *Γ* is the material tensile strength. Hence, the fitting slope in Figure 13 represents the material tensile strength *Γ*. The calculated tensile strengths of silver, copper, silver tin oxide and silver nickel alloy are 192, 217, 241 and 248 MPa, respectively.

### 4.4. Validity of Calculation Model

**Figure 14:**
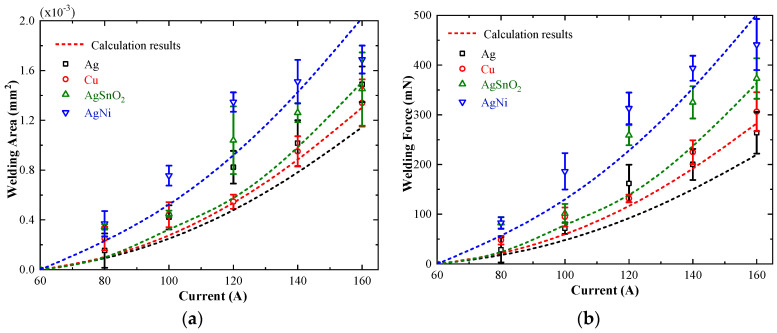
Comparison between the calculated values and experimental values for Ag, Cu, AgSnO_2_ and AgNi. (**a**) Welding area. (**b**) Welding force.

The variations in experimental results and calculation results of welding area as a function of load current for silver, copper, silver tin oxide and silver nickel alloy material are plotted in Figure 14a. Further, when the tensile strength of material and welding area is known, the welding force could also be calculated with the help of Equation (15) and the variations of welding force as a function of load current are shown in Figure 14b.

As shown, the welding area and associated welding force increase approximately in square with the load current. It is obvious that the calculation results of silver, copper, silver tin oxide and silver nickel alloy are very close to, but less than the experimental results over the entire range of load currents. The difference between the calculation results and experimental results may be caused by the simplification of our built model, in which the contact area is equivalent to a single circular *a*-spot. However, for all solid materials, the contact surface is rough on the micro scale, and there are multiple discrete contact points on the surface, and the real contact area is appreciably smaller than that of nominal contact. The small contact area is always accompanied by large heat production, resulting in a severe welding phenomenon, as mentioned above. Therefore, the calculation results are slightly lower than the experimental ones, especially for the weak elastic deformation situation.

The differences between the data groups can be evaluated by the value of the residual sum of squares (RSS), which is given as follows:

(16) $$\mathrm{RSS}=\sum_{i=1}^{n}{\left(A_{i}-B_{i}\right)}^{2}$$

where *A_i_* and *B_i_* are experimental results and calculation results, respectively. The smaller the RSS value is, the closer the calculation results are to the experimental results. Cosine similarity (COS) can be used to calculate the angle between two groups of data, which represents the similarity of the variation trend, and the closer the COS is to 1, the more similar the variation trend of the two groups of data is:

(17) $$\mathrm{COS}=\frac{\sum_{i=1}^{n}A_{i}\times B_{i}}{\sqrt{\sum_{i=1}^{n}{\left(A_{i}\right)}^{2}}\times \sqrt{\sum_{i=1}^{n}{\left(B_{i}\right)}^{2}}}$$

Taking the data of welding area as an example, the residual sum of squares (RSS) and cosine similarity (COS) between the calculation results and the experimental results are calculated to quantitatively analyze the accuracy of the mathematical model, and the calculation results are shown in Table 3. As shown, the value of the residual sum of squares (RSS) for silver, copper, silver tin oxide and silver nickel alloy are in the order of 10^−7^. The value of RSS for copper is the smallest, only 0.68 × 10^−7^, indicating that the calculation result of copper is the closest to the experimental result, which agree well with the results in Figure 14. Meanwhile, the value of the cosine similarity (COS) for each material is greater than 0.96, which means that the variation trend of the calculation results is highly consistent with that of the experimental results. The results indicate that the theoretical model is suitable to quantitatively analyze the welding area and welding force for contact materials.

## 5. Conclusions

A novel model for calculating the threshold welding current and welding area of metal electrode materials within low current switching devices has been developed and validated. The contact radius and temperature distribution have been studied by the elastic deformation theory and the heat transfer theory. The mechanical load force and load current both play important roles in material welding phenomena. Contact pairs with stronger mechanical load force require higher threshold load currents for producing the material welding phenomena. The reduced mechanical contact radius or load force could enlarge the welding area.

Experimental tests have been carried out on the silver, copper, silver tin oxide and silver nickel electrode materials to validate the model. The welding force and welding area increase with the increasing load current. The agreement between the calculated and measured data has been proved to be high with different materials. However, the calculation error due to the real rough surface of electrode materials could not be neglected. Moreover, the results demonstrate the silver material has the best anti-welding ability, owing to its low electrical resistivity, high thermal conductivity and small tensile strength. Silver nickel alloy is the easiest to welding electrode material. Investigation of the evaluation of material welding occurred in the switching process would be the future work.

## Figures and Tables

**Table 1:** Physical properties [5,21].

	*U_m_* (V)	*ρ*_0_ (μΩ·cm)	*α* (10^−3^/K)	*λ* (W/m·K)	*T*_1_ (K)	*E* (GPa)
Ag	0.37	1.59	4.1	419	1234	79
Cu	0.44	1.65	4.3	394	1356	115
AgSnO_2_	0.57	2.7	3.1	325	1233	86
AgNi	0.37	2.3	3.5	310	1233	84
Au	0.43	2.19	4	297	1336	80
Al	0.3	2.65	4.6	222	933	65
Zn	0.17	5.92	4.2	113	693	96
Ni	0.65	6.84	6.8	92	1726	216
Fe	0.6	9.72	6.6	75	1810	208
Sn	0.13	11.6	4.6	63	505	47

**Table 2:** Experimental conditions.

Environment	Ambient Temperature
Electrode Material	Ag, Cu, AgSnO_2_, AgNi
Load Current	60–160 A
Load Force	50 mN
Resistance	0.1 Ω

**Table 3:** Comparison between the calculation results and experimental results.

	RSS	COS
Ag	3.47 × 10^−7^	0.994
Cu	0.68 × 10^−7^	0.992
AgSnO_2_	4.75 × 10^−7^	0.96
AgNi	3.88 × 10^−7^	0.974
